# A Low-Power Optoelectronic Receiver IC for Short-Range LiDAR Sensors in 180 nm CMOS

**DOI:** 10.3390/mi15091066

**Published:** 2024-08-23

**Authors:** Shinhae Choi, Yeojin Chon, Sung Min Park

**Affiliations:** 1 Division of Electronic & Semiconductor Engineering, Ewha Womans University, Seoul 03760, Republic of Korea; rora0414@ewhain.net (S.C.); wjsdulws7@gmail.com (Y.C.); 2 Graduate Program in Smart Factory, Ewha Womans University, Seoul 03760, Republic of Korea

**Keywords:** A2V, APD, CMOS, LiDAR, optoelectronic, sensors, T2V

## Abstract

This paper presents a novel power-efficient topology for receivers in short-range LiDAR sensors. Conventionally, LiDAR sensors exploit complex time-to-digital converters (TDCs) for time-of-flight (ToF) distance measurements, thereby frequently leading to intricate circuit designs and persistent walk error issues. However, this work features a fully differential trans-impedance amplifier with on-chip avalanche photodiodes as optical detectors so that the need of the following post-amplifiers and output buffers can be eliminated, thus considerably reducing power consumption. Also, the combination of amplitude-to-voltage (A2V) and time-to-voltage (T2V) converters are exploited to replace the complicated TDC circuit. The A2V converter efficiently processes weak input photocurrents ranging from 1 to 50 μA_pp_ which corresponds to a maximum distance of 22.8 m, while the T2V converter handles relatively larger photocurrents from 40 μA_pp_ to 5.8 mA_pp_ for distances as short as 30 cm. The post-layout simulations confirm that the proposed LiDAR receiver can detect optical pulses over the range of 0.3 to 22.8 m with a low power dissipation of 10 mW from a single 1.8 V supply. This topology offers significant improvements in simplifying the receiver design and reducing the power consumption, providing a more efficient and accurate solution that is highly suitable for short-range LiDAR sensor applications.

## 1. Introduction

Light detection and ranging (LiDAR) sensors have been exploited in various fields, including advanced driver assistance systems, remote sensing detection and navigation systems, and short-range monitoring systems [1,2,3,4,5]. It is fairly well known that the amplitude-modulate-continuous-wave scheme estimates the detection range by the phase changes between the emitted (a.k.a. START) and received (a.k.a. STOP) pulses, while the frequency-modulated-continuous-wave method calculates the range using the frequency variations. Although the former acquires high precision for short distances and provides high resolution, it faces a number of disadvantages such as severe pulse distortions due to the multiple reflections from obstacles, limited detection range, and very complex algorithms for signal processing. The latter provides high precision and resolution, enables velocity measurements using the Doppler effect, and maintains a high signal-to-noise ratio (SNR). However, it requires high-performance computers to process the large volume of data, thus leading to substantially longer response times. In addition, the system implementation can be very complicated [6,7]. On the contrary, the pulsed time-of-flight (ToF) mechanism shows less precision and lower SNR and is more easily affected by environments such as fog, rain, and snow. Nonetheless, the ToF sensors are simple to design, which lowers the implementation cost, and makes them versatile for various applications. Therefore, this ToF scheme is preferred in this paper owing to its simplicity and low-cost characteristics.

**Figure 1:**
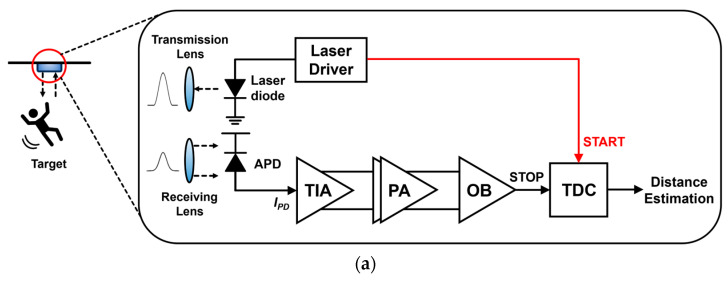

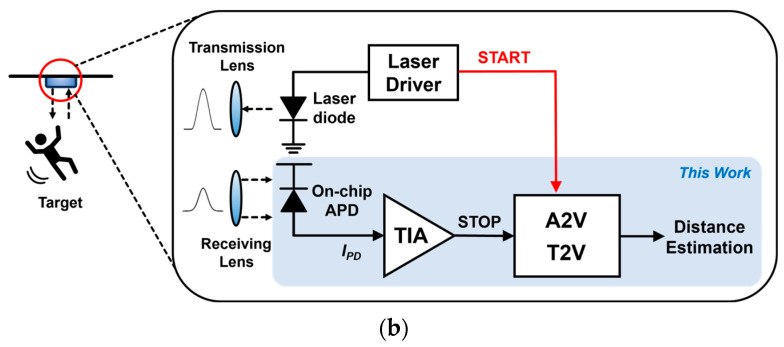
Block diagrams of (**a**) a conventional LiDAR sensor, and (**b**) the proposed LiDAR sensor.

Figure 1a shows a block diagram of a conventional short-range LiDAR sensor, where the transmitter is typically designed to be a laser diode driver so that light pulses can be transmitted to targets. Then, the reflected lights can be detected by the built-in receiver that consists of an optical photodetector (i.e., mostly avalanche photodiodes or APDs) to convert the incoming light pulses to electrical current signals, a trans-impedance amplifier (TIA) to transform the current signals to voltage outputs, a post-amplifier (PA) to boost the output voltages further, and an output buffer (OB) to isolate the preceding analog front-end (AFE) circuits from the following time-to-digital converter (TDC) that generates final digital codes to estimate the detection range. For the past few decades, these LiDAR sensors have advanced very fast but are mostly bulky and costly. Therefore, many efforts were conducted to achieve higher integration density and efficiency [7,8,9,10]. Particularly with TDC circuits, which are the most complicated and power-hungry blocks among the receivers.

Previously, various TDCs were suggested to accurately measure the time interval between the START and STOP pulses [11,12,13]. Yet, most TDCs mandate complicated methods and algorithms, and, therefore, the corresponding circuit designs could be very difficult to satisfy the specified requirements. Also, TDCs aim to reduce walk errors that usually occur at the inherent comparators in TDCs. Nonetheless, the inherently finite rising (or falling) edges of the STOP pulses could not remove the walk errors completely even with novel state-of-the-art TDCs.

In this paper, we propose a simpler and less complicated receiver topology for the applications of short-range LiDAR sensors. Figure 1b depicts the block diagram of the proposed LiDAR receiver, in which a fully differential TIA is newly proposed to omit the following PA and OB to achieve low power consumption. Also, the combination of analog-to-voltage (A2V) and time-to-voltage (T2V) converters is exploited to replace the complicated TDC circuitry. More specifically, the A2V converter transforms small input photocurrents ranging from 1 to 50 μA_pp_ linearly into output voltages and then holds the peak values until the next reset signal enters. Therefore, the A2V converter is highly effective in detecting weak signals reflected from targets located up to 22.8 m away. Nonetheless, it cannot process signals larger than 50 μA, which corresponds to a distance of 3.22 m.

Therefore, in order to enhance the dynamic range, especially for large input currents, the T2V converter takes the role of converting the time intervals between the START and STOP signals into output voltages. Then, the T2V converter holds the peak values until the next reset signal arrives. Simulations show that the proposed T2V converter can handle the large input currents from 40 μA_pp_ to 5.8 mA_pp_, which corresponds to the minimum detection range of 30 cm. Hence, the proposed LiDAR receiver can successfully detect the optical pulses within the range of 0.3 to 22.8 m.

This paper is organized as follows. Section 2 describes the circuit operations of the proposed LiDAR receiver along with the realization of on-chip P^+^/N-well/Deep N-well APDs. Section 3 presents the chip layout and the post-layout simulation results. Then, a conclusion follows in Section 4.

## 2. Circuit Description

### 2.1. On-Chip P^+^/NW/DNW APD

Typically, optical photodetectors are an off-chip component in optical receivers. However, the inevitable bond wires between the photodetector and the receiver chip cause several design issues including severe signal distortions. Moreover, the length of the bond wires cannot be controlled accurately and, therefore, the receiver performance would be quite different from what was anticipated. Furthermore, on-chip electro-static discharge (ESD) protection diodes are required to avoid damage from acute ESD. However, the ESD protection diodes give rise to additional parasitic capacitance, thus leading to bandwidth shrinkage. Hence, we realized on-chip photodetectors in this work to avoid the aforementioned problems. Particularly, APDs are preferred to p-i-n photodiodes despite their noisy characteristic because the targets are usually located within a few-meter distance, thus generating large photocurrents.

**Figure 2:**
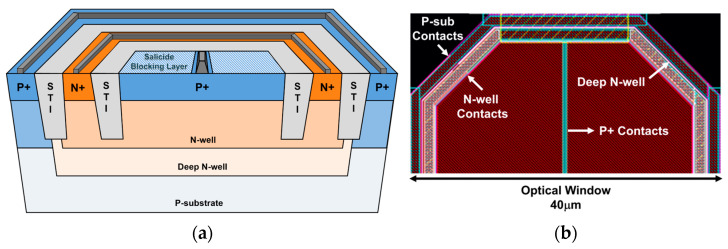
(**a**) A cross-sectional view of a P^+^/NW/DNW APD, (**b**) the layout of the on-chip APD.

Figure 2a illustrates the cross-sectional view of the developed on-chip CMOS P^+^/N-well/Deep N-well (or P^+^/NW/DNW) APD, where the avalanche multiplication is initialized by a hole occurred at the P^+^/NW junction. The shallow trench insulators (STIs) are inserted to prevent the possible premature edge breakdown. Also, the DNW layer is added to improve the near-infrared (NIR) sensitivity because the DNW layer decreases the number of holes spreading into the p-substrate. Particularly, the built-in potential barrier between the DNW and the p-substrate helps to exclude the photocurrents generated in the p-substrate.

Figure 2b shows the layout of the on-chip CMOS P^+^/NW/DNW APD, in which the octagonal shape is exploited to minimize the possible damage from the edge breakdown. It is noted that the P^+^ source and drain regions are covered by a blocking layer to form an optical window whereas the P^+^ contacts in the middle are unblocked because the salicidation process reduces the contact’s resistivity [14]. The diagonal length of the optical window is designed to be 40 µm so that the on-chip APD provides the −3-dB bandwidth of 1.7 GHz at a reverse bias of 10.25 V with a parasitic capacitance (C_PD_) of 470 fF.

### 2.2. Fully Differential Transimpedance Amplifier (FD-TIA)

**Figure 3:**
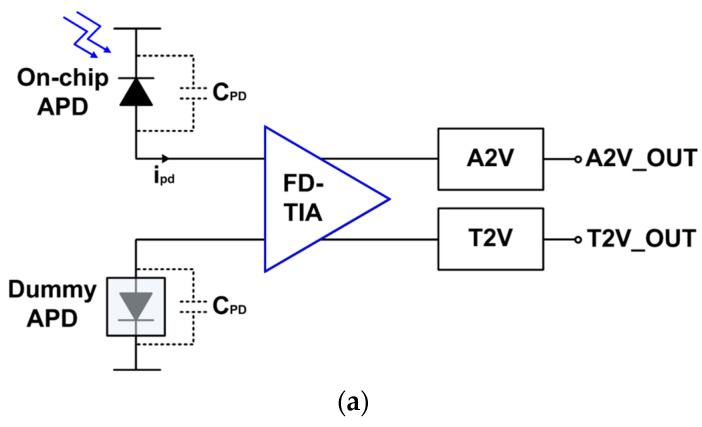

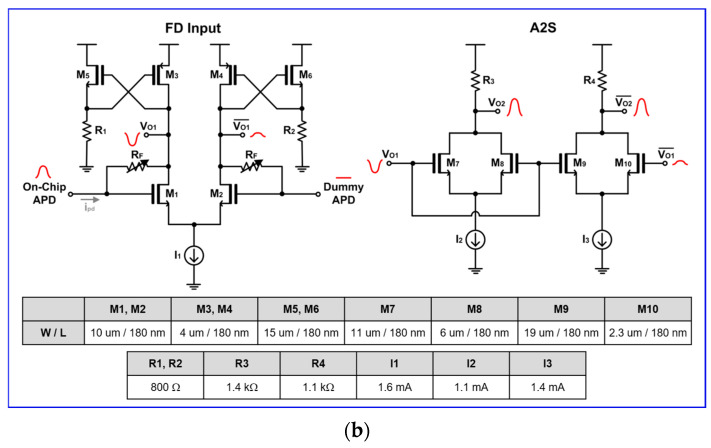
(**a**) Block diagram of the suggested LiDAR receiver and (**b**) schematic diagram of the proposed FD-TIA.

Figure 3a depicts the block diagram of the proposed LiDAR receiver, which consists of an on-chip P^+^/NW/DNW APD for optical-to-electrical conversion, another on-chip dummy P^+^/NW/DNW APD for input symmetry, and the proposed FD-TIA for current-to-voltage conversion, an A2V converter for weak signal recovery, and a T2V converter for large current detection. In addition, the FD-TIA includes an asymmetric-to-symmetric (A2S) converter together with the FD input stage, so that the symmetry of output waveforms can be improved further.

Figure 3b shows the schematic diagram of the FD-TIA in detail, where the FD-TIA incorporates a PMOS load (M_3_, M_4_) with a cross-coupled NMOS source-follower (M_5_ and R_1_, M_6_ and R_2_), allowing the output voltages to swing up to the supply voltage (V_DD_). Therefore, the incoming light pulses are converted to electrical current signals (*i_pd_*) by the on-chip APD. Then, the current signals flow towards the FD-TIA and are transformed to voltage outputs at the drain nodes of M_1_ and M_2_, respectively, by the action of the PMOS loads with cross-coupled NMOS source followers. Nonetheless, the output voltages at the drain nodes of the FD input stage cannot be perfectly matched due to the single-ended input signals. Hence, an A2S conversion circuit is proposed, as depicted in Figure 3b.

The A2S converter receives the output voltages of the FD-TIA and then generates more symmetric output signals since a larger output from the drain of M_1_ is connected to M_7_ and M_9_ simultaneously. Then, it results in symmetric output signals with a gain deviation of 0.6 dB at the drain nodes of M_7_~M_10_, as illustrated in Figure 3b.

In short, the A2S block can be effective in converting the asymmetric outputs to symmetric ones, and hence yields appropriate signals for both A2V and T2V converters. It should be noted that this conversion is acquired through the adjustment of resistors and transistor sizes in the A2S circuit. Furthermore, variable feedback resistors are added as an automatic-gain-control (AGC) circuit to vary the trans-impedance gain, thereby helping to extend the input dynamic range and enabling the T2V converter to operate better.

### 2.3. A2V Converter

**Figure 4:**
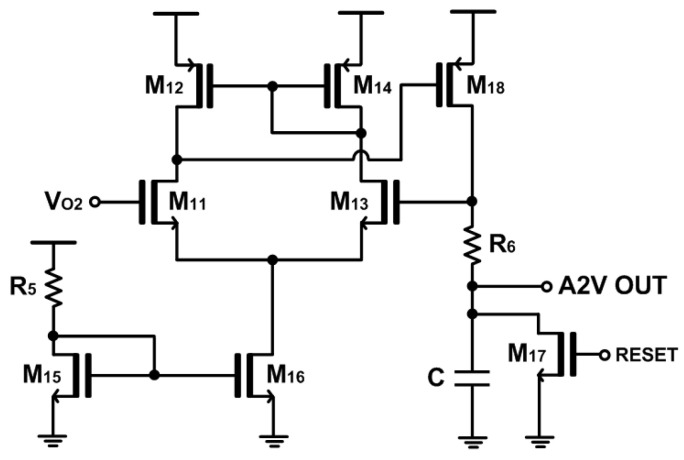
Schematic diagram of the peak detect and hold (PDH) for A2V conversion.

Figure 4 shows the schematic diagram of the proposed A2V converter that is simply a peak detect and hold (PDH) circuit. Its topology shares the basic architecture of a two-stage operational amplifier with the notable exception that the load is a series combination of a resistor (R_6_) and a capacitor (C) for the purpose of peak detection. The PDH circuit is designed to provide output pulses that are almost linearly proportional to the amplitudes of the incoming photocurrents. Namely, the main goal of this A2V converter is to enhance the detection capabilities of LiDAR sensors for targets situated at comparatively longer distances. Thereafter, a thermometer-to-binary circuit can be added to generate N-bit binary codes, which facilitates the post-processing of the following FPGA board.

### 2.4. T2V Converter

**Figure 5:**
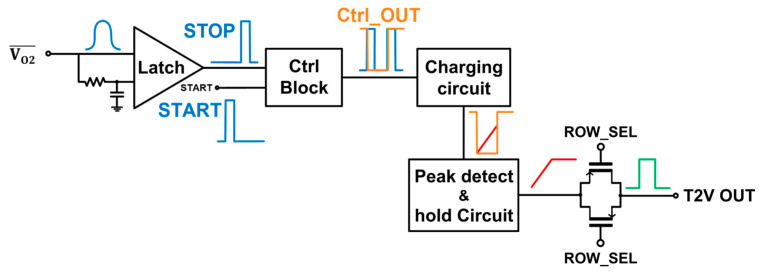
Block diagram of the proposed T2V converter.

Figure 5 shows the block diagram of the proposed time-to-voltage (T2V) converter that utilizes the integral method to extend the input dynamic range of the LiDAR receiver since it can convert the time-of-flight (ToF) information into the corresponding output pulses. Initially, the photocurrents generated by the on-chip CMOS P^+^/NW/DNW APD are converted into voltage signals via the FD-TIA and these voltage signals are then fed into the A2V converter.

Figure 5 also illustrates the signal flow in the proposed T2V converter, in which the output signals from the A2S converter of the FD-TIA are directly connected to the input latch. Then, the latch converts the incoming signals into digital pulses. Specifically, the latch yields an output ‘1’ when a signal is detected, while an output ‘0’ is generated when no signal is present. This latch output is subsequently fed into the control block together with the START signal that is transmitted from the transmitter (Tx) in advance. The control block provides an output ‘0’ for the duration between the START pulse and the received STOP pulse. Once the latch output pulse is detected, the control block switches the output to ‘1’, i.e., V_DD_.

**Figure 6:**
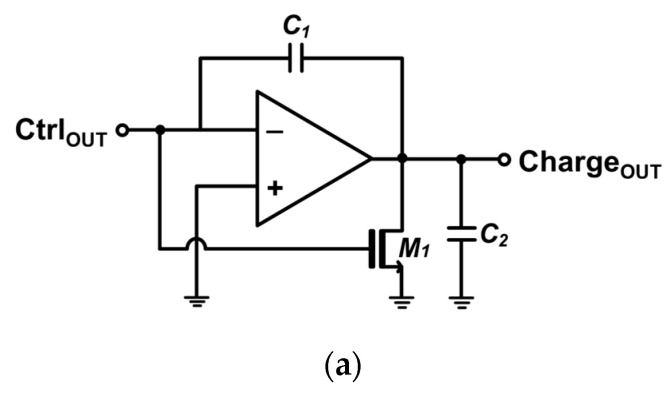

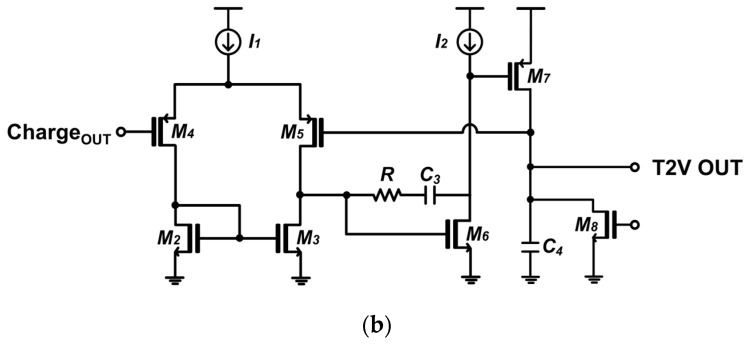
Schematic diagrams of the (**a**) charging circuit, and (**b**) peak detect and hold (PDH) circuit.

Thereafter, the output of the control block is fed into a charging circuit that can linearly increase its output voltage if the control block’s output remains ‘0’, forming a triangular waveform. Thus, the longer the time interval between the transmitter START signal and the received STOP signal is, the longer the output of the charging circuit rises. Hence, this T2V method allows precise and predictable range detection to be acquired by directly correlating the time-of-flight with the output voltage. Once the control block switches its output back to ‘1’, indicating signal arrival at the receiver, the charging circuit halts the voltage increment and maintains the peak voltage. Figure 6a depicts the schematic diagram of the charging circuit in the T2V converter.

Figure 6b shows the schematic diagram of the exploited peak detect and hold (PDH) circuit, which preserves the peak voltage of the charging circuit during the charging period until the next reset signal enters.

Finally, the peak voltage of the PDH circuit passes through a transmission gate (TG) that serves as the last stage of the T2V path. In summary, the proposed T2V converter efficiently converts the time-of-flight information into the proportional voltage signal. Each block of the T2V converter plays a critical role in ensuring precise and reliable conversion for accurate range detection. It should be noted that a thermometer-to-binary circuit can be added to this T2V converter circuit to generate N-bit binary codes, which facilitates the post-processing of the following FPGA board, as well.

## 3. Chip Layout and Post-Layout Simulation Results

The post-layout simulations were conducted utilizing the model parameters of a standard 180 nm CMOS process (TSMC, Hsinchu, Taiwan). In these simulations, the on-chip APDs were emulated as the equivalent electrical lumped model, which included a series resistance of 25 Ω and a parasitic capacitance of 490 fF.

**Figure 7:**
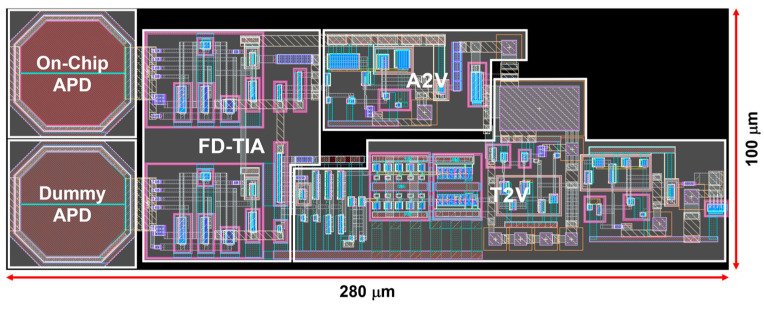
Layout of the proposed LiDAR receiver.

Figure 7 illustrates the layout of the proposed LiDAR receiver integrated with two on-chip P^+^/N-well/Deep N-well APDs, which occupy a core area of 100 × 280 μm^2^. DC simulations reveal that the proposed LiDAR receiver consumes 10 mW from a single 1.8 V supply.

**Figure 8:**
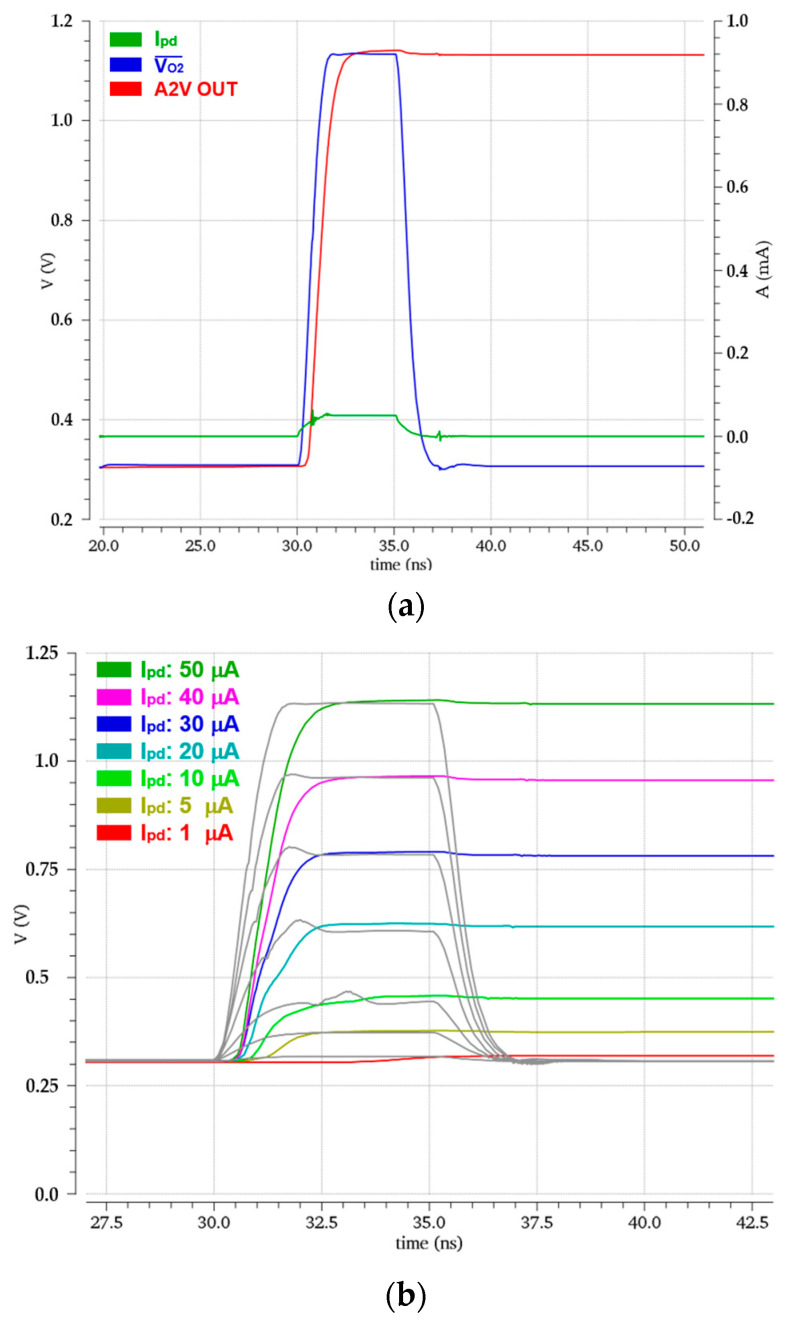
Simulated pulse responses of (**a**) the FD-TIA and the A2V converter, and (**b**) the A2V converter with the variations of the input currents.

Figure 8a shows the simulated pulse responses of the LiDAR receiver for a weak input current (*i_pd_*) of 50 µA_pp_ generated from the APD, where the FD-TIA generates 1.12 V_pp_ output voltage and the PDH circuit of the A2V converter maintains the peak voltage.

Figure 8b also shows the simulated transient responses with the variations of the input currents from 1 μA_pp_ to 50 μA_pp_ through the AGC scheme. These results correspond to the input dynamic range of 34 dB, allowing the maximum detection range of 22.8 m.

**Figure 9:**
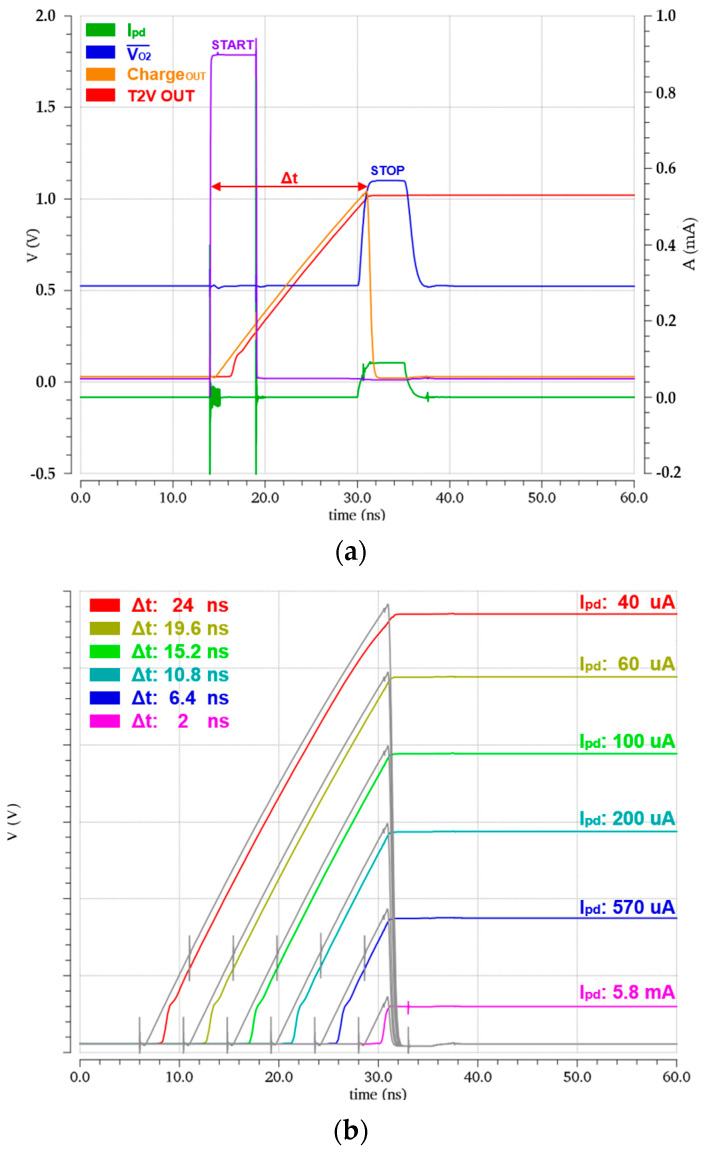
Simulated pulse response of (**a**) the FD-TIA and the T2V converter, and (**b**) the T2V converter with the variations of the time interval.

Figure 9a illustrates the simulated pulse responses of the LiDAR receiver for a large input current (*i_pd_*) of 90 µA_pp_ generated from the APD, where the FD-TIA provides 1.1 V_pp_ output voltage, the charging circuit yields the linearly increased output with the peak value of 1.02 V_pp_, and the PDH circuit of the T2V converter maintains the peak voltage. These simulation results confirm that the time intervals between the START signal emitted from the Tx and the received STOP signal can be detected and processed precisely by the T2V converter.

Figure 9b displays the simulation results with the variations of the input currents, demonstrating that the T2V converter can recover the input currents ranging from 40 μA_pp_ to 5.8 mA_pp_. These results correspond to the input dynamic range of 43.2 dB, allowing the detection of targets as close as 30 cm. It is clearly seen that the T2V converter can effectively detect the large input current levels that the A2V converter alone cannot process, thereby indicating their complementary operations between these two converters.

Table 1 lists the performance deviations resulting from the process, voltage, and temperature (PVT) variations for three worst-case scenarios: (**a**) SS, 1.62 V, −45 °C, (**b**) TT, 1.8 V, 27 °C, and (**c**) FF, 1.98 V, 125 °C, respectively. Here, it is seen that the trans-impedance (TZ) gain variation of the proposed FD-TIA is less than 4.1%, the bandwidth deviates by less than 1.43% from the nominal 842 MHz, and the noise current spectral density fluctuates by less than 22%. The transient response of the A2V converter shows that the output voltage swing changes within 14% for the input currents below 50 μA_pp_. Also, the T2V converter output varies within 28% for the input currents above 40 μA_pp_ with a time interval (ΔT) of 24 ns. These results certainly prove the stable operations of the proposed LiDAR receiver.

Table 2 compares the performance of the proposed LiDAR receiver with prior arts. Ref. [15] realized a CMOS receiver-TDC chip set that successfully demonstrated superior performance, in which the receiver chip consisting of an off-chip APD, a TIA, a post-amplifier, and two timing-comparators, achieved a wide dynamic range of 92 dB, while the multi-channel TDC chip comprising delay-lock loops, time digitizers, and oscillator acquired a timing resolution of less than 10 ps and the maximum detection range of 80 m. However, these two chips occupied a very bulky area and dissipated quite a large amount of power (or DC bias current).

Ref. [16] suggested a low-complexity hybrid readout circuit, i.e., one sampling and storage array with an embedded TDC that could provide both timing and amplitude information simultaneously. In particular, a narrow bandwidth TIA (89 MHz in simulations) was exploited to reduce noise. Yet, an equalizer with built-in threshold voltage was necessary to expand the bandwidth, thereafter, leading to a relatively large current consumption. Also, the maximum detection current was unfortunately limited to 2.06 mA_pp_ only.

Ref. [17] presented an eight-channel AFE circuit with two operational modes for the purpose of power saving, which consisted of reconfigurable TIAs, variable gain amplifiers, OBs, and mode-selector circuits for either parallel mode or selectable mode. Also, two different photodetectors, i.e., a p-i-n photodiode for high-gain mode and an APD for low-gain mode, were utilized for gain switching. However, the necessity of two photodetectors might cause high cost and integration difficulties on the PC board. Moreover, the channel switchover time was ~10 ns in selectable mode, which might cause signal loss since the incoming pulse width is typically less than 10 ns. Moreover, the dynamic range was considerably narrow when compared to other works.

Ref. [18] demonstrated a ToF receiver where an off-chip LC resonator was combined with a shunt-feedback TIA for fast response, a non-linear feedback circuit was exploited in the TIA by using auxiliary transistors for low input resistance, four-stage cascaded post-amplifiers were followed with a Gm-C integrator feedback for efficient DC offset cancellation, and timing comparators with regenerative latch and a differential-to-single operational amplifier were incorporated to result in final pulses. Although the ToF receiver achieved a very wide dynamic range due to the non-linear feedback even with the absence of AGC circuitry, the chip dissipated relatively large power (or DC bias current) from the utilized 3.3 V supply. Also, an additional TDC chip was required to finalize the data. In addition, the off-chip APD mandated a very large bias voltage (below 140 V), and the utilized LC resonator might highly increase the integration cost.

Therefore, this work shows competent performance despite the low responsivity of the on-chip CMOS APD and the comparatively low trans-impedance gain characteristics. In particular, it achieves a moderately wide dynamic range with the aid of the proposed A2V and T2V converters. More importantly, it provides very low power dissipation and small chip area characteristics because it can discard power-hungry post-amplifiers and a complex TDC circuit, therefore, enabling it to provide a feasible solution for low-cost, low-power LiDAR sensors.

**Table 2:** Performance comparison with the recently reported LiDAR sensors.

Parameters		[15]	[16]	[17]	[18]	This Work
CMOS technology (nm)		350	180	180	350	180
APD	Type	Off-chip	^‡^ Off-chip	^‡^ Off-chip	Off-chip	On-chip
	C_pd_ (pF)	3	2	3	4	0.5
	Responsivity (A/W)	40	-	-	-	4.16
	Wavelength (nm)	905	-	-	905	850
Max. TZ gain (dBΩ)		100	83.5	100	121	81.2
Gain control		No	Yes	Yes	No	Yes
Bandwidth (MHz)		230	89	180	230	842
Min. detectable current (µA_pp_)		1.0	1.3	5	0.6	1.0
Max. detectable current (mA_pp_)		39 ^ξ^	2.06	2	30	5.8
Dynamic range (dB)		92	64	52	94	75.3
Power dissipation per channel (mW) (DC bias current consumption)		330 * (100 mA)	45 * (13.6 mA)	49.3/10.5 (27.4/5.83 mA)	155 * (47 mA)	10 (5.56 mA)
FoM ^ψ^		70	165	365	180	6837
Chip area (mm^2^)		4 (Rx 1ch) 10 (TDC)	2.52 (8ch ^†^)	5 (Rx 8ch)	2.89 (Rx 1ch)	1 (Rx 3ch)

^ξ^ with a separate TDC chip, * 3.3 V V_DD_, ^†^ ch = channel, ^‡^ equivalent circuit model. ^ψ^ FoM = TZ gain × bandwidth/power dissipation.

## 4. Conclusions

We have presented a power-saving receiver topology for short-range LiDAR sensors, where the proposed LiDAR receiver exploits an FD-TIA to eliminate the need for power-hungry post-amplifiers and output buffers, thereby reducing power consumption significantly. Also, the LiDAR receiver incorporates A2V and T2V converters to replace complex TDC circuitry. The A2V converter effectively detects weak input photocurrents ranging from 1 to 50 mA_pp_, reflected from the targets located up to 22.8 m away. Since it is limited to signals below 50 mA_pp_ which correspond to a distance of 3.22 m, the T2V converter is employed in parallel to handle the large input currents. The T2V converter facilely translates the time intervals between the START and STOP signals into the corresponding output voltages by handling currents from 40 mA_pp_ to 5.8 mA_pp_, which allows for a minimum detection range of 30 cm. The post-layout simulations confirm that the proposed LiDAR receiver can accurately detect optical pulses within the range of 0.3 to 22.8 m with a power dissipation of 10 mW from a single 1.8 V supply. Hence, this topology offers a promising solution for low-power, low-cost, short-range LiDAR sensors.

## Figures and Tables

**Table 1:** PVT variation simulations of the proposed LiDAR receiver including the FD-TIA.

Parameters		SS 1.62 V, −45 °C	TT 1.8 V, 27 °C	FF 1.98 V, 125 °C
FD-TIA	TZ gain (dBΩ)	84.5 (+4.1%)	81.2	77.9 (−4.1%)
	Bandwidth (GHz)	839 (−0.36%)	842	854 (+1.43%)
	$\mathrm{Noise}\;\mathrm{current}\;\mathrm{spectral}\;\mathrm{density}\;(\mathrm{pA}/\sqrt{\mathrm{Hz}}$)	8.67 (−14%)	10.1	12.3 (+22%)
A2V	Output voltage amplitude (mV_pp_) @ 1 µA_pp_ input current	327 (+2.2%)	320	343 (+7.2%)
	Output voltage amplitude (V_pp_) @ 50 µA_pp_ input current	0.96 (−14%)	1.12	1.27 (+13%)
T2V	Output voltage amplitude (V_pp_) @ 40 µA_pp_ (Δt = 24 ns) input current	1.16 (−19%)	1.43	1.72 (+20%)
	Output voltage amplitude (mV_pp_) @ 5.8 mA_pp_ (Δt = 2 ns) input current	106 (−27%)	146	187 (+28%)

## Data Availability

Data are contained within the article.
